# The Association between Early Childhood and Later Childhood Sugar-Containing Beverage Intake: A Prospective Cohort Study

**DOI:** 10.3390/nu11102338

**Published:** 2019-10-01

**Authors:** Andrea Ziesmann, Ruhi Kiflen, Vanessa De Rubeis, Brendan T. Smith, Jonathon L. Maguire, Catherine S. Birken, Laura N. Anderson, on behalf of the TARGet Kids Collaboration

**Affiliations:** 1 Department of Health Research Methods, Evidence, and Impact, McMaster University, Hamilton, ON L8S 4L8, Canada; ziesmana@mcmaster.ca (A.Z.); ruhikiflen27@gmail.com (R.K.); derubevg@mcmaster.ca (V.D.R.); 2 Department of Health Promotion, Chronic Disease and Injury Prevention, Public Health Ontario, Toronto, ON M5G 1V2, Canada; Brendan.Smith@oahpp.ca; 3 Dalla Lana School of Public Health, University of Toronto, Toronto, ON M5T 3M7, Canada; jonathon.maguire@utoronto.ca (J.L.M.); catherine.birken@sickkids.ca (C.S.B.); 4 Applied Health Research Centre of the Li Ka Shing Knowledge Institute of St. Michael’s Hospital, University of Toronto, Toronto, ON M5G 1B1, Canada; 5 Department of Nutritional Sciences, Faculty of Medicine, University of Toronto, Toronto, Ontario M5S 1A8, Canada; 6 Department of Pediatrics, St. Michael’s Hospital, Toronto, ON M5C 2T2, Canada; 7 Division of Child Health Evaluative Sciences (CHES), Sick Kids Research Institute, Toronto, ON M5G 0A4, Canada

**Keywords:** sugars, fruit juices, life-course epidemiology, infant, child

## Abstract

Sugar-containing beverages (SCBs) are a major source of sugar intake in children. Early life intake of SCBs may be a strong predictor of SCB intake later in life. The primary objective of this study was to evaluate if SCB intake (defined as 100% fruit juice, soda, and sweetened drinks) in early childhood (≤2.5 years of age) was associated with SCB intake in later childhood (5–9 years of age). A prospective cohort study was conducted using data from the TARGet Kids! primary care practice network (*n* = 999). Typical daily SCB intake was measured by parent-completed questionnaires. Odds ratios (OR) and 95% confidence intervals (CI) were estimated using logistic regression. A total of 43% of children consumed ≥0.5 cups/day of SCBs at ≤2.5 years and this increased to 64% by 5–9 years. Daily SCB intake, compared to no daily intake, at ≤2.5 years was significantly associated with SCB intake at 5–9 years (adjusted OR: 4.03; 95% CI: 2.92–5.55) and this association was much stronger for soda/sweetened drinks (adjusted OR: 12.83; 95% CI: 4.98, 33.0) than 100% fruit juice (OR: 3.61; 95% CI: 2.63–4.95). Other early life risk factors for SCB intake at 5–9 years were presence of older siblings, low household income, and shorter breastfeeding duration. Daily intake of SCBs in early childhood was strongly associated with greater SCB intake in later childhood. Early life may be an important period to target for population prevention strategies.

## 1. Introduction

Sugar intake in children has been associated with increased cardiometabolic risk factors, including obesity [1,2], high blood pressure, and dyslipidemia [3,4,5,6,7,8] and dental caries [9,10]. These adverse conditions in childhood are associated with disease outcomes in later adulthood, such as type 2 diabetes and cardiovascular disease [11,12,13]. Further, sugar intake across the life-course is associated with similar adult disease outcomes [2,14]. In 2015, fruit juice and sweetened fruit drinks were among the top ten most common sources of sugar intake for children ages two to eight in Canada, amounting to approximately 11.6% and 3.5% of their total sugar intake, respectively [15]. In the US, it was found that children and adolescents derive 10%–15% of total calories from sugar-containing beverages [16]. Recent estimates suggest that the percentage of U.S. children 19–23 months of age consuming any 100% fruit juice is about 50%, and 38% consumed any sugar-sweetened beverages [17].

Sugar-sweetened beverages are frequently defined as liquids that are sweetened with various forms of added sugars (e.g., sodas, fruit drinks) [18]. Fruit juices which contain 100% juice are often excluded from the definition of sugar-sweetened beverages, as they are sometimes perceived as a healthier beverage option. However, 100% fruit juice also contains a high sugar content and lacks dietary fibre. The World Health Organization defines free sugars as “all monosaccharides and disaccharides added to foods and beverages by the manufacturer, cook or consumer, as well as sugars that are naturally present in honey, syrups, fruit juices and fruit juice concentrates”, and recommends that both adults and children reduce their intake of free sugars to less than 10% of total energy intake [19]. The American Academy of Pediatrics recommends limiting children’s fruit juice intake [20], which is consistent with the newly released 2019 Canada Food Guide, which also suggests limiting, if not replacing sugar-containing drinks with water [21]. The term sugar-containing beverages (SCBs) is used here to include the following beverages containing free sugars: Soda or pop, sweetened drinks, and 100% fruit juice.

Dietary patterns in childhood have been shown to track into adulthood [22,23], thus the early introduction of sugar-sweetened beverages may increase intake across the life-course [24,25,26]. Studies conducted in the United States [26] and Norway [25] reported that intake of soda and sweetened beverages before two years of age is strongly associated with increased intake in later childhood at age six and seven, respectively. However, neither study included 100% fruit juice, and only one study controlled for potential confounders [26]. Further, a recent systematic review of risk factors associated with sugar beverage intake in children <6 years of age also did not include 100% fruit juice intake in its definition of SCB [27]. This systematic review identified 17 determinants of sugar beverages that support an association with intake of SCB in early childhood, including child age, parental knowledge, household rules, screen time, snack consumption, formula fed, lower parental socioeconomic status, and early introduction of solids [10,27]. Few studies have prospectively evaluated SCB intake during infancy and risk factors in early life that may predict SCB intake in later childhood.

The primary objective of this study was to evaluate if SCB intake in early childhood (≤2.5 years of age) was associated with increased SCB intake in later childhood (5–9 years of age). The secondary objective was to evaluate if the association between early life SCB intake and later childhood intake was independent of other established SCB risk factors.

## 2. Materials and Methods

### 2.1. Study Design

A prospective cohort study was conducted among children in The Applied Research Group for Kids (TARGet Kids!) primary care practice-based research network. The TARGet Kids! network recruits children <6 years of age at scheduled well-child visits and follows them prospectively through childhood [28]. Children were recruited between 2008 and 2017 from primary care pediatricians or family practice clinics in the Greater Toronto Area, Canada. Children were excluded at enrollment if they had associated health conditions affecting growth (i.e., failure to thrive, cystic fibrosis), severe developmental delay, or other chronic conditions except for asthma or if the families were not English speaking. Informed written consent was obtained from parents and TARGet Kids! is approved by the research ethics boards at the Hospital for Sick Children (#1000012436) and St. Michael’s Hospital, Toronto, Canada.

### 2.2. Study Population and Sample Size

**Figure 1:**
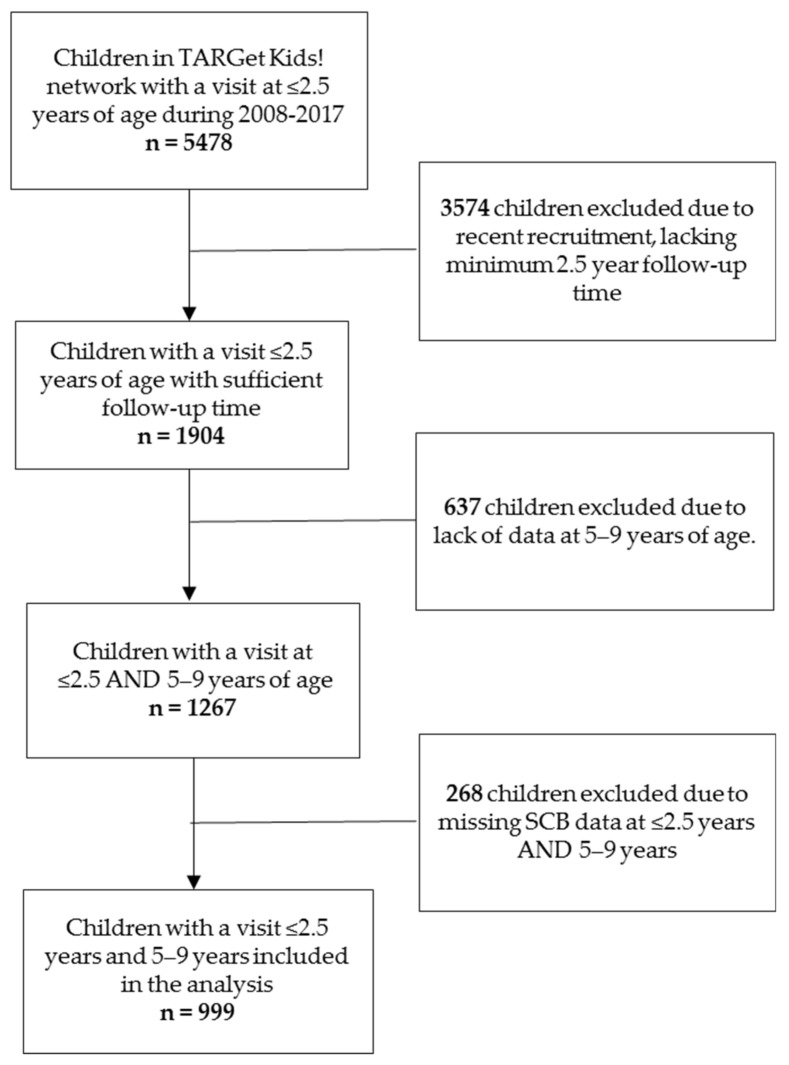
Participant flow diagram.

For this study, children were included if they had at least one visit before or at 2.5 years of age (defined as early childhood) and a follow-up at 5–9 years of age (defined as later childhood) from 2008 to 2017. A total of 5478 children were identified in the TARGet Kids! database who had a visit at ≤2.5 years of age, 3574 children were excluded because they were recruited recently and would not have a minimum of 2.5 year follow-up time (i.e., these children had not yet reached 5 years of age). Of the remaining 1904 children who had a visit at ≤2.5 years of age there were 1267 with a follow-up visit between 5 and 9 years of age. However, 268 children were missing data on SCB intake at both time points (≤2.5 years of age, and 5–9 years of age) and therefore were removed, resulting in a total sample size of 999 children (Figure 1).

### 2.3. Measurement of Sugar-Containing Beverage Intake

At each TARGet Kids! visit, parents completed an age-specific nutrition and health questionnaire [28]. To determine a child’s sugar beverage intake, parents were asked to “circle how many cups of each drink your child has currently in a typical day, if none then circle 0 (1 cup = 8 ounces = 250 mL)” and the response options were: 0, 1/2, 1, 2, 3, 4, and 5+. The list of beverages included “100% juice (apple, orange etc.)”, “sweetened drinks (Kool Aid, Sunny D, etc.)” and “soda or pop”. In this analysis, three primary exposure variables were investigated: (1) 100% fruit juice, (2) soda and sweetened drinks (combined sweetened drinks with soda/pop), and (3) total sugar-containing beverages (SCBs), which includes all three beverages (100% fruit juice, soda, and sweetened drinks). Similar to previous studies in this age group [26] all variables were dichotomized into 0 cups/day and ≥0.5 cups/day since a high proportion of respondents indicated no intake.

### 2.4. Other Variables

Based on a recent systematic review of risk factors for sugar beverage intake in children <6 years of age [27], we identified variables of interest associated with SCB consumption, including age, sex, mother’s education, mother’s ethnicity, family income, number of siblings, zBMI, weekday free time play, parental BMI, and length of breastfeeding. Child and parent BMI were measured through the measurement of height and weight by trained research assistants using standard methods [29]. Child overweight and obesity categories were defined using BMI z-scores calculated using the WHO growth reference standard which is recommended for child growth monitoring in Canada [30]. All other variables were captured through questionnaires completed by parents.

### 2.5. Statistical Analysis

All statistical analyses were conducted using SAS software 9.4 (Raleigh, NC, USA). Descriptive analyses for all variables were conducted, including the frequency and percent, and when applicable, the mean and standard deviation. To assess whether the intake of SCBs in early childhood (ages ≤2.5) was associated with intake in later childhood (ages 5–9), multivariable logistic regression was used to estimate odds ratios (OR) and corresponding 95% confidence intervals (CI). Multivariable models included all potential risk factors identified a priori based on a previous systematic review. Results are presented for three dependent variables at 5–9 years of age: (1) total SCB intake; (2) sugar and sweetened drink; and (3) 100% fruit juice. All variables were adjusted for as presented in Table 1. Multicollinearity was assessed by inspection of the variance of inflation factors (VIFs). None of the VIFs were greater than 2.5, suggesting no concerns with multicollinearity. To account for missing data in the covariates, multiple imputation was performed using PROC MI and PROC MIANALYZE on SAS. Participants who were missing data on both the primary exposures and outcome variables were excluded from the study thus these variables were not imputed, only data on covariates was imputed. A minimum of 20 datasets were imputed for each analysis. All variables listed in Table 2 were included in the imputation model, including the exposure (≤2.5 years of age) and outcome (5–9 years of age). All statistical tests were 2-sided and a significance level of less than 0.05 was specified.

## 3. Results

A total of 999 children had measures of sugar-containing beverage intake between 0 and 2.5 years of age and 5 and 9 years of age (Figure 1). Baseline demographic characteristics are described in Table 1. Approximately 46.7% of children were female, 73.0% were of European ethnicity, and the mean age at the first visit was 18.1 months. There were 141 children (14.1%) who met the WHO defined cut-points for ‘risk of overweight’ and 34 (3.4%) who were ‘overweight or obese’ based on their zBMI at ≤2.5 years of age.

### 3.1. Description of SCB Consumption at ≤2.5 Years and 5–9 Years

**Figure 2:**
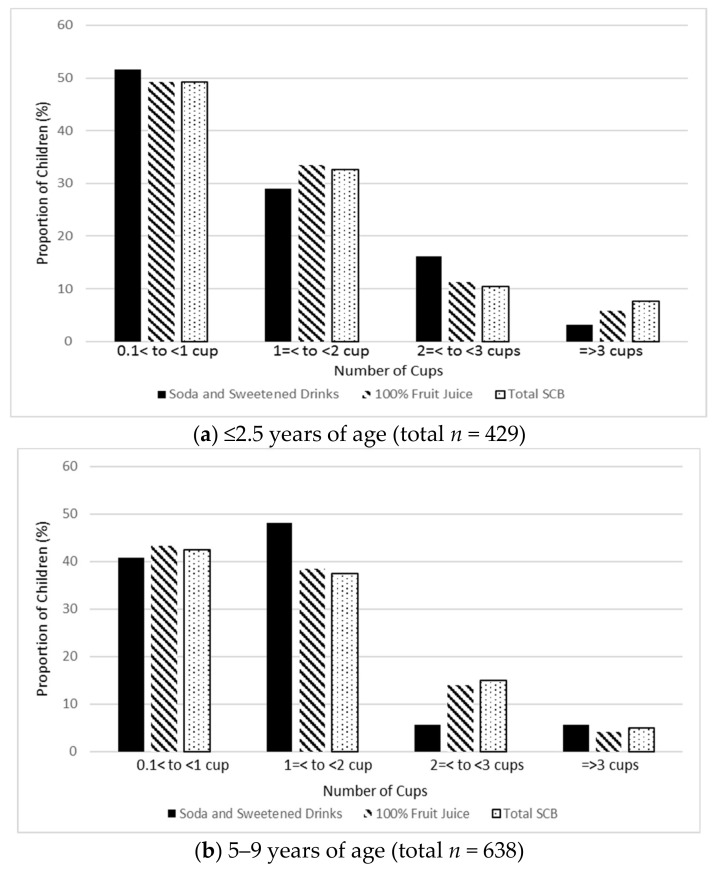
Distribution of sugar-containing beverage (SCB) intake among children in the TARGet Kids! Research Network among children who “ever consumed” SCBs.

The proportion of children that typically drank ≥0.5 cups/day of 100% fruit juice was 42.9% at ≤2.5 years of age, which increased to 62.9% at ages 5–9 (Table 2). At ≤2.5 years of age, only 3.2% of children typically consumed ≥0.5 cups/day of soda and sweetened drinks, which increased to 6.1% of children at 5–9 years of age. At ≤2.5 years, 57.1% of children consumed 0 cups/day of SCB, which decreased to 36.1% at ages 5–9. This represents a 21% decrease in those who consumed 0 cups/day of SCB. At ≤2.5 years of age, the majority of children (51.6%) who consumed soda and sweetened drinks, drank 0 to 1 cups (Figure 2a), whereas at 5–9 years of age, almost half of children (48.1%) drank 1 to 2 cups of soda and sweetened drinks (Figure 2b). Among children who consumed any SCB, 49.2% consumed 0 to 1 cups at ≤2.5 years of age (Figure 2a), and 42.5% consumed this amount at 5–9 years of age (Figure 2b).

### 3.2. SCB Intake at ≤2.5 Years Association with SCB Intake at 5–9 Years

A strong and significant association was observed between SCB intake at ≤2.5 years of age and later in childhood (5–9 years) and this persisted in the fully adjusted model after controlling for potential SCB risk factors (Table 3). For total SCB intake, children who consumed ≥0.5 cups/day of SCBs at ≤2.5 years old had 4.03 times greater odds of consuming SCBs in later childhood (fully adjusted OR: 4.03; 95% CI: 2.92–5.55) (Table 3). Children who had consumed soda and sweetened drinks at ≤2.5 years of age had 16.91 times greater odds of consuming soda and sweetened drinks at ages 5–9 (fully adjusted OR:16.91; 95% CI: 3.68, 77,69) (Table 3). Those who consumed ≥0.5 cups/day of 100% fruit juice at ≤2.5 years of age had 3.6 times greater odds of consuming fruit juice at ages 5–9 (fully adjusted OR: 3.61; 95% CI: 2.63–4.95) (Table 3). The associations for 100% fruit juice and total SCB changed minimally in the models adjusted only for age and sex versus the fully adjusted models (Table 3). However, the association for soda and sweetened drinks changed substantially with an OR of 23.48 (95% CI: 9.55, 57.76) for the model adjusted only for age and sex, and this decreased to 16.91 (95% CI: 3.68, 77.69) for the fully adjusted model (Table 3).

### 3.3. Other Early Childhood Risk Factors

Other possible risk factors for SCB consumption in later childhood (5–9 years of age) are also presented in Table 3. For total SCB intake, breastfeeding decreased odds of consuming SCBs in later childhood (fully adjusted OR: 0.94; 95% CI: 0.89, 0.99 per two months longer breast feeding) (Table 3). Having a household income of <$49,999 (fully adjusted OR: 1.64; 95% CI: 0.79, 3.39), in comparison to a household income ≥$150,000, was suggestive of increased odds of consuming SCBs at 5–9 years, although not statistically significant. Significant risk factors for increased odds of soda and sweetened drink intake included children with one sibling in comparison to having no siblings (fully adjusted OR: 3.72; 95% CI, 1.20, 11.47); a mother of East, South, or South-East Asian ethnicity in comparison to European (fully adjusted OR: 7.39; 95% CI: 2.47, 22.11); a household income of <$49,999 (fully adjusted OR: 11.96; 95% CI: 2.86, 50.09) in comparison to a household income ≥$150,000; parental BMI (per 5 kg/m^2^) increase (OR: 1.09; 95% CI: 1.01, 1.18). For every two-month increase of breastfeeding duration there was a 0.89 reduction of odds of consuming soda and sweetened drink consumption when adjusting for all potential risk factors (95% CI: 0.82, 0.97) (Table 3). No variables were statistically significant risk factors for the consumption of 100% fruit juice at 5–9 years of age (Table 3), but, consistent with total SCB consumption, the fully adjusted ORs were greater than 1.0 for household income <$49,999 (fully adjusted OR: 1.67; 95% CI: 0.82, 3.40), and having a mother of East, South, or South-East Asian ethnicity (fully adjusted OR: 1.37; 95% CI: 0.88, 2.12).

## 4. Discussion

In this study 100% fruit juice, soda, and sweetened drinks, and total SCB intake at ≤2.5 years of age, were each strongly associated with higher SCB intake at 5 –9 years of age. These associations were observed after adjustment for other potential early life risk factors of SCB intake. The findings from this study contribute evidence to the current literature supporting the association of SCB intake in later childhood when SCBs are introduced in early childhood. These results are consistent with two previous longitudinal cohort studies from the United States [26] and Norway [25]. The U.S. study included 1333 children with data at <1 and at 6 years of age. They found that children with intake of sugar-sweetened beverages at any time during infancy, compared to no intake during infancy, had 2.22 times higher odds for consuming sugar-sweetened beverages at least once per day at 6 years of age (OR: 2.22; 95% CI: 1.59-3.10) [26]. The Norwegian study included 9025 children participating at three time points (18 months, 36 months, and 7 years). They observed that children with low, medium, and high frequency of sugar-sweetened beverage intake at 18 months continued to drink sugar-sweetened beverage just as often at 36 months and 7 years of age [25]. However, these studies only examined the intake of soda and sweetened drinks and excluded 100% fruit juice. To the best of our knowledge, no previous studies have investigated 100% fruit juice in early childhood as a risk factor for later childhood SCB consumption. Understanding the impact of early childhood consumption of SCBs is imperative, as dietary patterns and behaviors have been found to influence behaviors throughout the life-course [31,32,33].

Following a recent systematic review that identified risk factors for SCB intake in children [27], we prospectively evaluated the associations between potential risk factors during early childhood (≤2.5 years of age) and later childhood (5–9 years of age) SCB intake, including 100% fruit juice, soda, and sweetened drinks, and total SCB intake (which included all three beverages). No statistically significant risk factors were identified for fruit juice intake and all associations evaluated were close to null, except for early life fruit juice intake. However, children were at greater odds of consuming soda and sweetened drinks if they had one sibling (compared to none), if maternal ethnicity was East, South, or South-East Asian (compared to European), if the household income was <$49,999 (compared to ≥$150, 000), and increasing parental BMI, with ORs suggesting 1.09- to 11.96-fold increased odds for each of these previously identified risk factors. Child BMI was not associated with any SCB intake, which may reflect the young age of our study population. However, parent BMI was significantly associated with increased odds of soda and sweetened drink intake among children, and although not statistically significant, the ORs were in the same direction for total SCB and fruit juice intake. Results of the analyses for total SCBs (combining fruit juice, soda, and sweetened drinks) only identified one significant risk factor, breastfeeding duration, and this is likely explained by the fact that 100% fruit juice was the major contributor to total SCBs.

The proportion of children who consumed SCBs in this study population may appear low, but is somewhat comparable to national data from the 2015 Canadian Community Health Survey (CCHS). In the 2015 CCHS for children 1–8 years of age [34], 46.1% consumed fruit juice, 6.3% of children consumed regular soft drinks, and 14.4% consumed fruit drinks. The 2017 American Academy of Pediatric recommendations for children’s juice intake indicate that infants <12 months of age should not be introduced to juice, children aged 1–3 should be limited to 4 oz. (0.5 cup) daily, children aged 4–6 should only consume 4–6 oz. daily, and children aged 7–18 should be limited to 8 oz. daily [20]. At ≤2.5 years of age, 21.9% of children from this sample were drinking more than the recommended limit of 4 oz. daily, and at 5–9 years of age 35.3% were drinking more than the recommended 4–6 oz. per day. The new 2019 Canada Food Guide suggests limiting, if not replacing sugar-containing drinks (e.g., fruit juice, soft drinks, sports drinks, fruit flavored drinks, and punches) with water, as there are many benefits to this practice [21]. These guidelines are general and not specific to age groups, perhaps to encourage the same healthy lifestyles throughout the life-course.

A potential limitation of this study is measurement error because of parent-reported questionnaires used to measure child SCB intake. However, studies have found that parent reported SCB is a valid measure of true SCB intake in children [35,36]. It is possible that parents misclassified 100% fruit juices as sweetened drinks or vice versa, however, regulations state that juices that are not 100% fruit juice must be identified as a juice “drink”, “beverage”, or “cocktail”, and thus should have been classified as a sweetened drink, not as 100% fruit juice [37,38]. Further, we did not collect data on all possible SCBs. For example, data were not available on sweetened milks or energy drinks. Infant formula was also not included within the analysis. Since a high proportion of respondents indicated no intake, all SCB variables were dichotomized into 0 cups/day and ≥0.5 cups/day for all analyses, which may contribute to a loss of power. Another potential limitation of this study is the missing data for SCB intake at ≤2.5 years and 5–9 years, and all covariates. To address this limitation, we applied a multiple imputation approach to account for the missing data and limit potential biases associated with missing data. In addition, the study had a follow-up response rate of 52%. The low follow-up rates contributed to a reduction in the sample size leading to lower certainty in the ability to make strong conclusions. Lastly, the sample of the current study may not be representative of all Canadian children, since TARGet Kids! is an urban primary care network with relatively high family income and education levels. Although it may be assumed that SCB consumption during early childhood is mainly influenced by parents, it is possible that other factors, such as school environment, may also play a role in consumption patterns, especially in older children. We were unable to evaluate these factors, but future studies may be able to further understand the role these factors play in defining the consumption patterns of SCBs in children. Strengths of this study include the prospective nature of the study design with comprehensive measurements of potential SCB risk factors in early life, including 100% fruit juice. Few studies have investigated SCB intake and risk factors in early childhood. However, this may be a sensitive period in development from both a biologic sugar exposure and behavioural development point of view. Literature shows that taste preferences are developed in the prenatal, neonatal, infancy, and early childhood stages of life [39,40]. Early introduction to sugary beverages is only one of the many biological, social, and environmental factors that continue to be influence taste preferences throughout the lifespan.

## 5. Conclusions

The results of this study suggest that SCB intake in early childhood is strongly associated with SCB intake in later childhood and this association persists even after adjustment for many other possible predictors of SCB intake. Future studies are needed to understand longer-term associations and to evaluate if interventions targeting SCB reduction in early life reduce both SCB intake and adverse health outcomes in later childhood, adolescence and adulthood.

## Figures and Tables

**Table 1:** Baseline characteristics of children from the TARGet Kids cohort with a visit at both ≤2.5 years old and 5–9 years old (*n* = 999).

Characteristic	*n*	%
Sex		
Male	533	53.4
Female	466	46.7
Maternal Education		
College/University	917	91.8
High school or Less	56	5.6
Missing	26	2.6
Number of Siblings		
0	442	44.2
1	378	37.8
≥2	107	10.7
Missing	72	7.2
Family Income ^1^		
<$49,999	65	6.5
$50,000-99,999	161	16.1
$100,000-149,999	173	17.3
≥$150,000	555	55.6
Missing	45	4.5
Child zBMI ^2^		
Normal and underweight (zBMI ≤ 1.0)	790	79.1
Risk of overweight (1 > zBMI ≤ 2.0)	141	14.1
Overweight and obesity (zBMI > 2)	34	3.4
Missing	34	3.4
Maternal Ethnicity		
European	729	73.0
East, South, or South-East Asian	135	13.5
Other	102	10.2
Missing	33	3.3
	**Mean**	**SD**
Age (months)	18.1	4.8
Parent BMI (kg/m^2^) ^3^	24.6	4.6
Typical Weekday Free Play (minutes)	58.7	56.5
Child zBMI	0.09	1.07
Breastfeeding duration (months)	11.3	5.7

^1^ Canadian dollars. ^2^ Based on WHO Growth Standards. ^3^ Responses were from either mothers (84%) or fathers (16%).

**Table 2:** Distribution of sugar-containing beverage consumption in children at ≤2.5 years of age and 5–9 years of age among children in TARGet Kids! in Ontario, Canada (*n* = 999).

Sugar-Containing Beverages	≤2.5 years of age N (%)	5–9 years of age N (%)
100% Fruit Juice ^1^		
0 cups/day	564 (57.1)	368 (37.1)
≥0.5 cups/day	424 (42.9)	623 (62.9)
Soda and Sweetened Drinks ^2^		
0 cups/day	934 (96.8)	829 (93.9)
≥0.5 cups/day	31 (3.2)	54 (6.1)
Total SCB consumption		
0 cups/day	570 (57.1)	361 (36.1)
≥0.5 cups/day	429 (42.9)	638 (63.9)

^1^ 11 missing 100% fruit juice intake at ≤2.5 years of age and 8 missing 100% fruit juice intake at 5–9 years of age; ^2^ 34 missing soda and sweetened drink intake at ≤2.5 years of age and 116 missing soda and sweetened drink intake at 5–9 years of age.

**Table 3:** Odds ratio estimates for total sugar-containing beverages (SCB), soda and sweetened drinks, and fruit juice intake at 5–9 years of age, for children with a visit at ≤2.5 years of age and 5–9 years in the TARGet Kids! Research Network (*n* = 999).

	*Minimally Adjusted Model* Total SCB Intake at 5–9 Years of Age		*Fully Adjusted Model* Total SCB Intake at 5–9 Years of Age		*Minimally Adjusted Model* Soda and Sweetened Drink at 5–9 Years of Age		*Fully Adjusted Model* Soda and Sweetened drink at 5–9 Years of Age		*Minimally Adjusted Model* Fruit Juice Intake at 5–9 Years of Age		*Fully Adjusted Model* Fruit Juice Intake at 5–9 Years of Age	
	OR ^1^	95% CI	OR ^2^	95% CI	OR ^1^	95% CI	OR ^2^	95% CI	OR ^1^	95% CI	OR^2^	95% CI
Total SCB at ≤2.5 years												
0 cups/day	1.00		1.00									
≥0.5 cups/day	**4.33**	**3.18, 5.91**	**4.03**	**2.92, 5.55**								
Soda & sweetened drinks at ≤2.5 years												
0 cups/day					1.00		1.00					
≥0.5 cups/day					**23.48**	**9.55, 57.76**	**16.91**	**3.68, 77.69**				
Fruit Juice at age ≤2.5 years												
0 cups/day									1.00		1.00	
≥0.5 cups/day									**3.85**	**2.83, 5.23**	**3.61**	**2.63, 4.95**
Age (months)	1.02	0.99, 1.05	0.98	0.95, 1.01	0.99	0.94, 1.06	0.98	0.87, 1.09	0.98	0.95, 1.00	0.98	0.95, 1.01
Sex												
Male	1.00		1.00		1.00		1.00		1.00		1.00	
Female	**1.33**	**1.01, 1.75**	0.77	0.58, 1.02	0.67	0.36, 1.23	0.43	0.65, 8.21	0.78	0.59, 1.02	0.80	0.60, 1.05
Education (mother)												
College/University			1.00				1.00				1.00	
Highschool or less			1.90	0.89, 4.05			2.31	0.69, 4.92			1.26	0.63, 2.52
Sibling								**1.20, 11.47** 0.54, 11.89				
0			1.00				1.00				1.00	
1			1.10	0.80, 1.50			**3.72**				1.15	0.85, 1.56
2+			1.14	0.71, 1.82			2.55				1.08	0.67, 1.75
Ethnicity (mother)								**2.47, 22.11** 0.43, 2.90				
European			1.00				1.00				1.00	
East, South or South-East Asian			1.34	0.86, 2.08			**7.39**				1.37	0.88, 2.12
Other			0.93	0.58, 1.50			1.12				0.85	0.53, 1.35
Household income				0.79, 3.39 0.89, 1.80 0.82, 1.80				**2.86, 50.09** 0.38, 4.79 0.41, 5.20				0.82, 3.40 0.90, 2.00 0.82, 1.76
<49,999			1.64				**11.96**				1.67	
50,000–99,999			1.33				1.16				1.34	
100,000–149,999			1.22				1.46				1.19	
≥150,000			1.00				1.00				1.00	
Child zBMI (per 1-unit increase)			1.04	0.91, 1.19			0.79	0.49, 1.27			1.04	0.91, 1.20
Breastfeeding duration (per 2 months increase)			**0.94**	**0.89, 0.99**			**0.89**	**0.82, 0.97**			0.95	0.91, 1.01
Parental BMI ^3^ (per 5-unit increase)			1.12	0.93, 1.34			**1.09**	**1.01, 1.18**			1.12	0.94, 1.33
Typical weekday free play (per 30 min increase)			1.04	0.96, 1.28			1.00	1.00, 1.01			1.03	0.95, 1.13

^1^ Adjusted for age and sex only. ^2^ Adjusted for all listed variables. ^3^ 84% of responses were collected from the mother and 16% were from fathers. Bolded text within the table represent statistically significant values (*p* < 0.05).

## References

[1] Frantsve-Hawley J., Bader J.D., Welsh J.A., Wright J.T. (2017). A systematic review of the association between consumption of sugar-containing beverages and excess weight gain among children under age 12. J. Public Health Dent..

[2] Hu F.B. (2013). Resolved: There is sufficient scientific evidence that decreasing sugar-sweetened beverage consumption will reduce the prevalence of obesity and obesity-related diseases. Obes. Rev..

[3] Vos M.B., Kaar J.L., Welsh J.A., Van Horn L.V., Feig D.I., Anderson C.A.M., Patel M.J., Cruz Munos J., Krebs N.F., Xanthakos S.A. (2017). Added Sugars and Cardiovascular Disease Risk in Children: A Scientific Statement From the American Heart Association. Circulation.

[4] Fidler Mis N., Braegger C., Bronsky J., Campoy C., Domellöf M., Embleton N.D., Hojsak I., Hulst J., Indrio F., Lapillonne A. (2017). Sugar in Infants, Children and Adolescents: A Position Paper of the European Society for Paediatric Gastroenterology, Hepatology and Nutrition Committee on Nutrition. J. Pediatr. Gastroenterol. Nutr..

[5] Ambrosini G.L., Oddy W.H., Huang R.C., Mori T.A., Beilin L.J., Jebb S.A. (2013). Prospective associations between sugar-sweetened beverage intakes and cardiometabolic risk factors in adolescents. Am. J. Clin. Nutr..

[6] Kell K.P., Cardel M.I., Bohan Brown M.M., Fernández J.R. (2014). Added sugars in the diet are positively associated with diastolic blood pressure and triglycerides in children. Am. J. Clin. Nutr..

[7] Kosova E.C., Auinger P., Bremer A.A. (2013). The relationships between sugar-sweetened beverage intake and cardiometabolic markers in young children. J. Acad. Nutr. Diet..

[8] Wang J.W., Mark S., Henderson M., O’Loughlin J., Tremblay A., Wortman J., Paradis G., Gray-Donald K. (2013). Adiposity and glucose intolerance exacerbate components of metabolic syndrome in children consuming sugar-sweetened beverages: QUALITY cohort study. Pediatr. Obes..

[9] Moynihan P.J., Kelly S.A. (2014). Effect on caries of restricting sugars intake: Systematic review to inform WHO guidelines. J. Dent. Res..

[10] Scientific Advisory Committee On Nutrition (2015). Carbohydrates and Health.

[11] Malik V.S., Popkin B.M., Bray G.A., Després J.P., Willett W.C., Hu F.B. (2010). Sugar-sweetened beverages and risk of metabolic syndrome and type 2 diabetes: A meta-analysis. Diabetes Care.

[12] Sahoo K., Sahoo B., Choudhury A.K., Sofi N.Y., Kumar R., Bhadoria A.S. (2015). Childhood obesity: Causes and consequences. J. Fam. Med. Prim. Care.

[13] Raitakari O.T., Juonala M., Kahonen M., Taittonen L., Laitinen T., Maki-Torkko N., Jarvisalo M.J., Uhari M., Jokinen E., Ronnemaa T. (2003). Cardiovascular risk factors in childhood and carotid artery intima-media thickness in adulthood: The Cardiovascular Risk in Young Finns Study. JAMA.

[14] Nissinen K., Mikkilä V., Männistö S., Lahti-Koski M., Räsänen L., Viikari J., Raitakari O.T. (2009). Sweets and sugar-sweetened soft drink intake in childhood in relation to adult BMI and overweight. The Cardiovascular Risk in Young Finns Study. Public Health Nutr..

[15] Langlois K., Garriguet D., Gonzalez A., Sinclair S., Colapinto C.K. (2019). Change in total sugars consumption among Canadian children and adults. Health Rep..

[16] Wang Y.C., Bleich S.N., Gortmaker S.L. (2008). Increasing caloric contribution from sugar-sweetened beverages and 100% fruit juices among US children and adolescents, 1988-2004. Pediatrics.

[17] Hamner H.C., Perrine C.G., Gupta P.M., Herrick K.A., Cogswell M.E. (2017). Food Consumption Patterns among U.S. Children from Birth to 23 Months of Age, 2009-2014. Nutrients.

[18] U.S. Department of Health and Human Services 2015–2020 Dietary Guidelines for Americans. https://health.gov/dietaryguidelines/2015/guidelines/.

[19] World Health Organization (2014). Guideline: Sugar Intake for Adults and Children.

[20] Heyman M.B., Abrams S.A., SECTION ON GASTROENTEROLOGY, HEPATOLOGY, AND NUTRITION, COMMITTEE ON NUTRITION (2017). Fruit Juice in Infants, Children, and Adolescents: Current Recommendations. Pediatrics.

[21] Health Canada (2019). Canada’s Dietary Guidelines 2019.

[22] Mikkilä V., Räsänen L., Raitakari O.T., Pietinen P., Viikari J. (2005). Consistent dietary patterns identified from childhood to adulthood: The cardiovascular risk in Young Finns Study. Br. J. Nutr..

[23] Movassagh E.Z., Baxter-Jones A.D.G., Kontulainen S., Whiting S.J., Vatanparast H. (2017). Tracking Dietary Patterns over 20 Years from Childhood through Adolescence into Young Adulthood: The Saskatchewan Pediatric Bone Mineral Accrual Study. Nutrients.

[24] Kvaavik E., Andersen L.F., Klepp K.I. (2005). The stability of soft drinks intake from adolescence to adult age and the association between long-term consumption of soft drinks and lifestyle factors and body weight. Public Health Nutr..

[25] Bjelland M., Brantsæter A.L., Haugen M., Meltzer H.M., Nystad W., Andersen L.F. (2013). Changes and tracking of fruit, vegetables and sugar-sweetened beverages intake from 18 months to 7 years in the Norwegian Mother and Child Cohort Study. BMC Public Health.

[26] Park S., Pan L., Sherry B., Li R. (2014). The association of sugar-sweetened beverage intake during infancy with sugar-sweetened beverage intake at 6 years of age. Pediatrics.

[27] Mazarello P., Hesketh K., O’Malley C., Moore H., Summerbell C., Griffin S., Ong K., Lakshman R. (2015). Determinants of sugar-sweetened beverage consumption in young children: A systematic review. Obes. Rev..

[28] Carsley S., Borkhoff C.M., Maguire J.L., Birken C.S., Khovratovich M., McCrindle B., Macarthur C., Parkin P.C., Collaboration T.K. (2015). Cohort Profile: The Applied Research Group for Kids (TARGet Kids!). Int. J. Epidemiol..

[29] Carsley S.E., Anderson L.N., Plumptre L., Parkin P.C., Maguire J.L., Birken C.S. (2017). Severe Obesity, Obesity, and Cardiometabolic Risk in Children 0 to 6 Years of Age. Child. Obes..

[30] De Onis M. (2006). WHO Child groth standards based on length/height, weight, and age. Int. J. Paediatr..

[31] Skinner J.D., Carruth B.R., Bounds W., Ziegler P., Reidy K. (2002). Do food-related experiences in the first 2 years of life predict dietary variety in school-aged children?. J. Nutr. Educ. Behav..

[32] Fiorito L.M., Marini M., Mitchell D.C., Smiciklas-Wright H., Birch L.L. (2010). Girls’ early sweetened carbonated beverage intake predicts different patterns of beverage and nutrient intake across childhood and adolescence. J. Am. Diet. Assoc..

[33] Teegarden D., Lyle R.M., Proulx W.R., Johnston C.C., Weaver C.M. (1999). Previous milk consumption is associated with greater bone density in young women. Am. J. Clin. Nutr..

[34] Garriguet D. (2019). Changes in beverage consumption in Canada. Health Rep..

[35] Lora K.R., Davy B., Hedrick V., Ferris A.M., Anderson M.P., Wakefield D. (2016). Assessing Initial Validity and Reliability of a Beverage Intake Questionnaire in Hispanic Preschool-Aged Children. J. Acad. Nutr. Diet..

[36] Lewis K.H., Skelton J.A., Hsu F.C., Ezouah P., Taveras E.M., Block J.P. (2018). Implementing a novel electronic health record approach to track child sugar-sweetened beverage consumption. Prev. Med. Rep..

[37] Clemens R., Drewnowski A., Ferruzzi M.G., Toner C.D., Welland D. (2015). Squeezing fact from fiction about 100% fruit juice. Adv. Nutr..

[38] Government of Canada Juice and Juice Products. http://www.inspection.gc.ca/food/requirements-and-guidance/labelling/industry/processed-fruit-or-vegetable-products/juice-and-juice-products/eng/1348153076023/1348153220895.

[39] Ventura A.K., Mennella J.A. (2011). Innate and learned preferences for sweet taste during childhood. Curr. Opin. Clin. Nutr. Metab. Care.

[40] Ventura A.K., Worobey J. (2013). Early influences on the development of food preferences. Curr. Biol..

